# Supplementary material for: Tetracistronic Minigenomes Elucidate a Functional Promoter for Ghana Virus and Unveils Cedar Virus Replicase Promiscuity for all Henipaviruses
Source: bioRxiv. 2024 Apr 16:2024.04.16.589704. Preprint. [Version 1] doi: 10.1101/2024.04.16.589704 (PMC11042316; doi:10.1101/2024.04.16.589704)
Supplement: Supplement 1 [file NIHPP2024.04.16.589704v1-supplement-1.pdf]

bioRxiv preprint doi: https://doi.org/10.1101/2024.04.16.589704; this version posted April 16, 2024. The copyright holder for this preprint (which was not certified by peer review) is the author/funder, who has granted bioRxiv a license to display the preprint in perpetuity. It is made available under aCC-BY 4.0 International license.

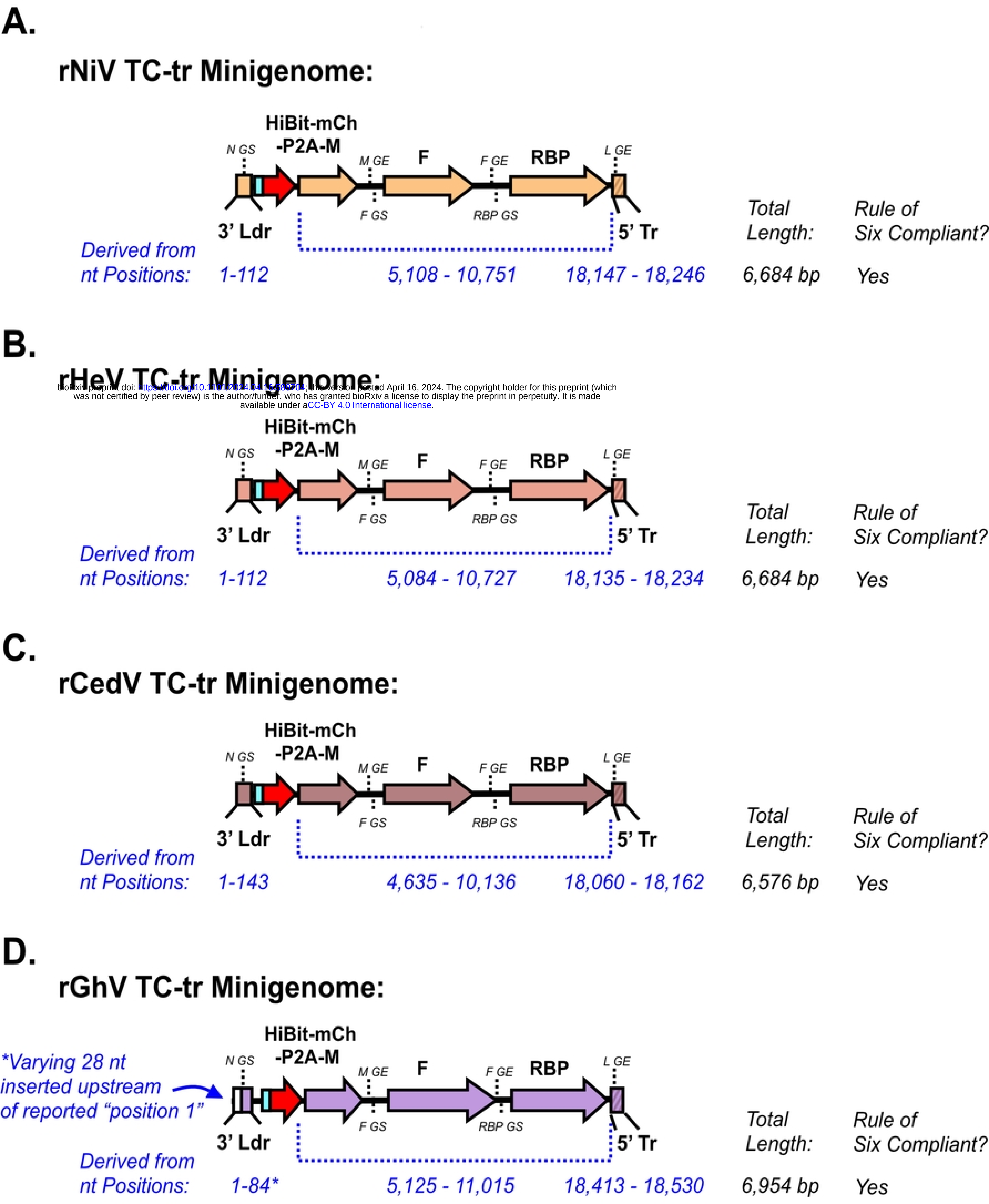

Supplementary Figure 1

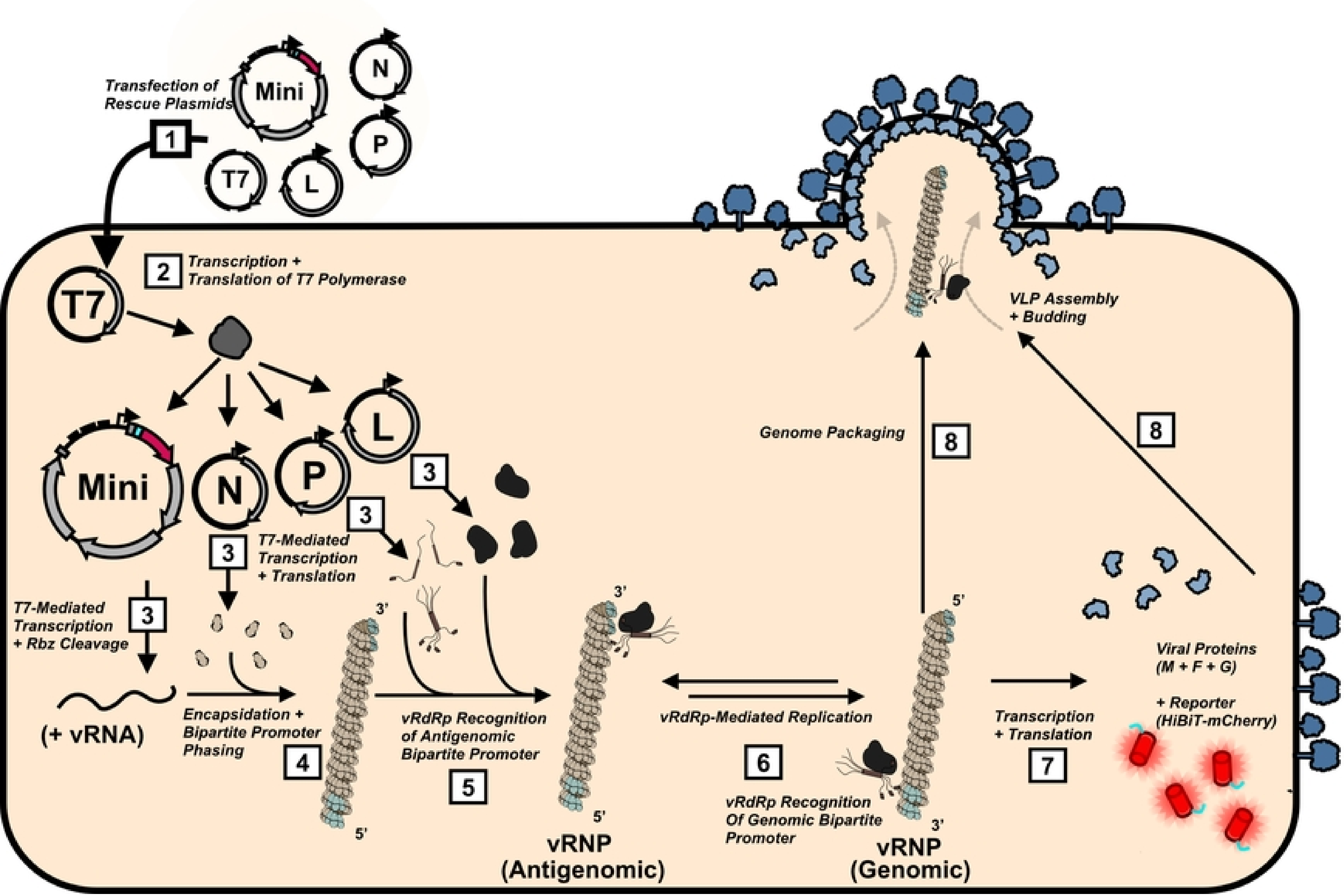

Supplementary Figure 2

bioRxiv preprint doi: https://doi.org/10.1101/2024.04.16.589704; this version posted April 16, 2024. The copyright holder for this preprint (which was not certified by peer review) is the author/funder, who has granted bioRxiv a license to display the preprint in perpetuity. It is made available under aCC-BY 4.0 International license.

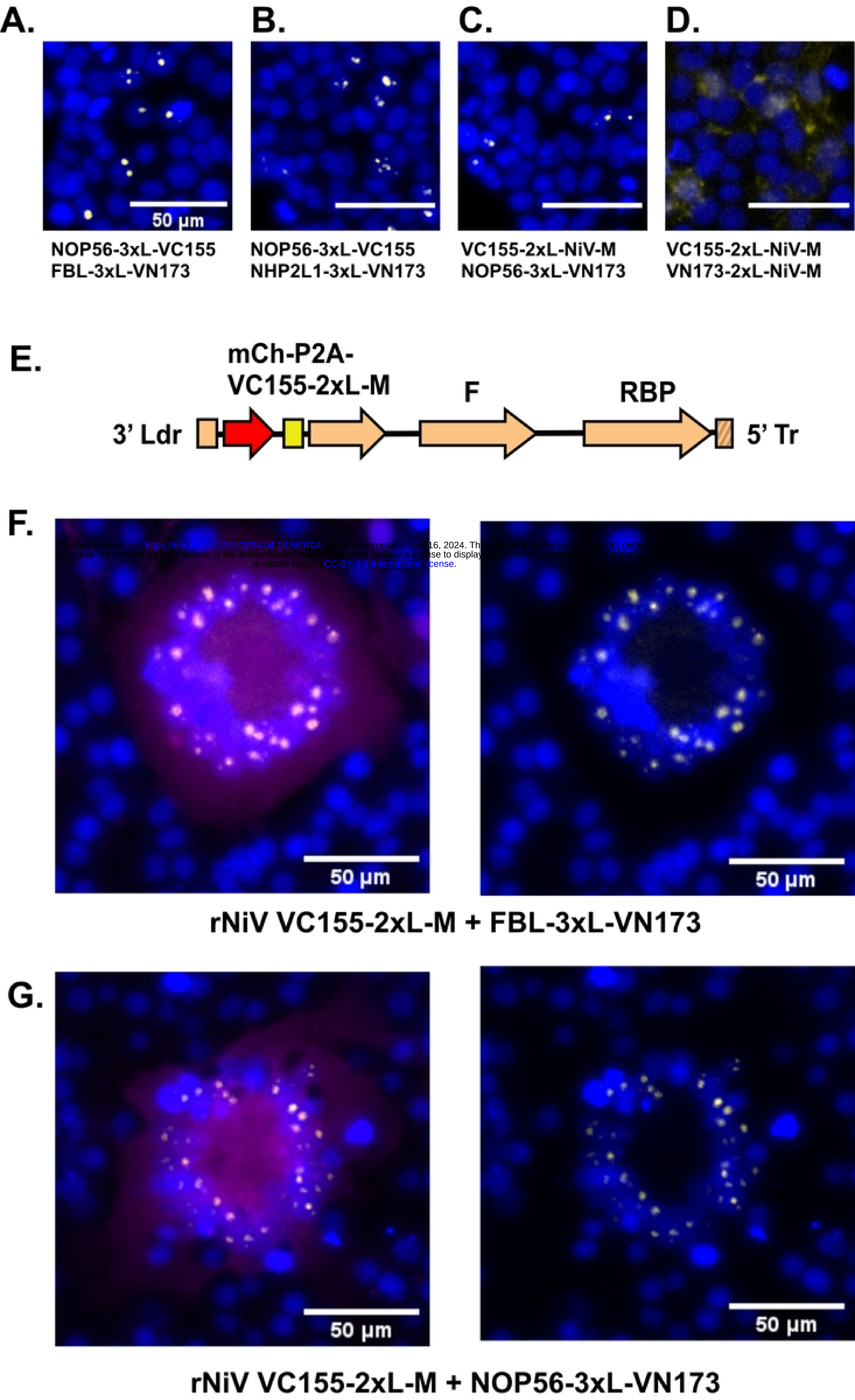

Supplementary Figure 3

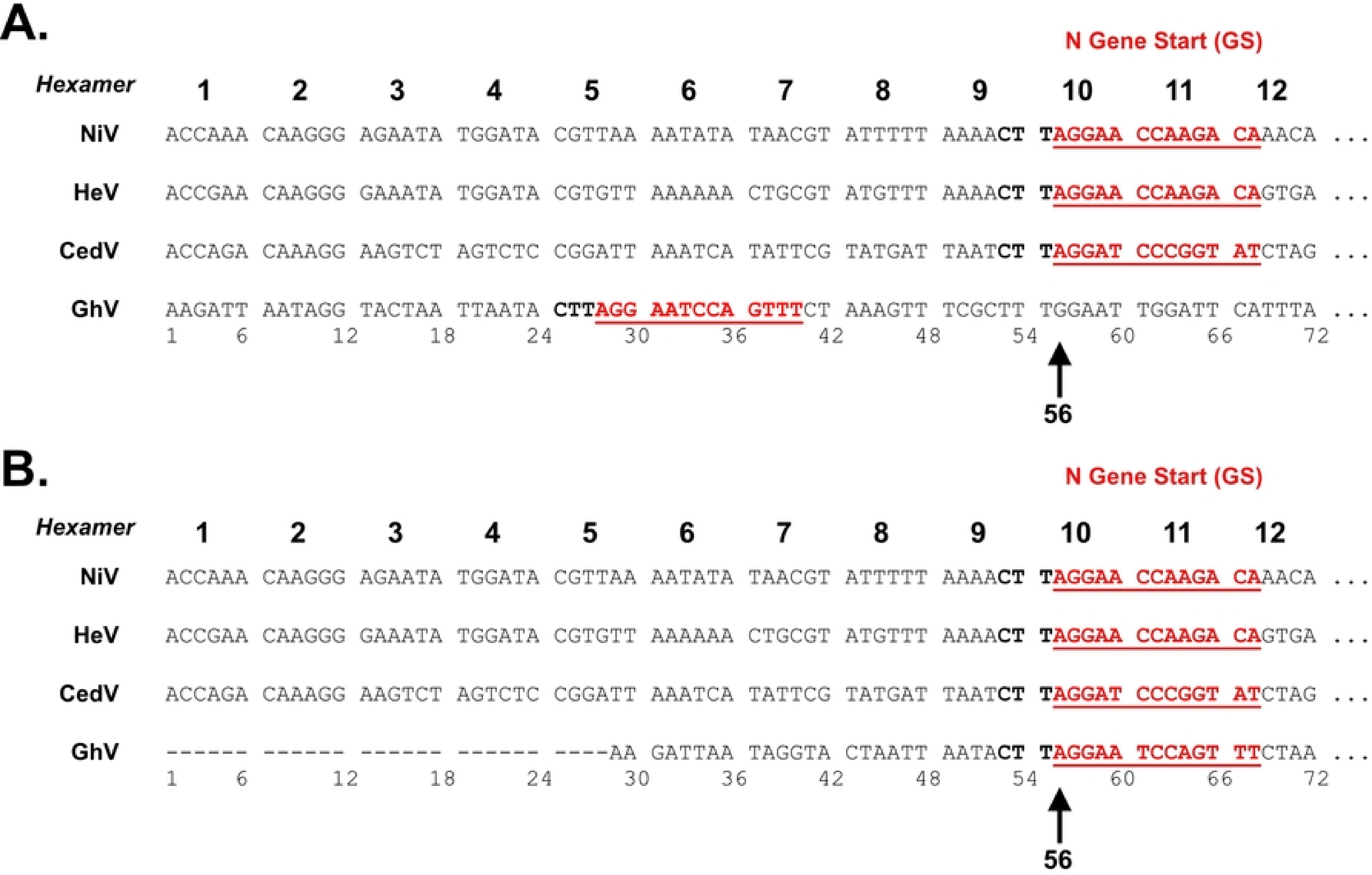

Supplementary Figure 4

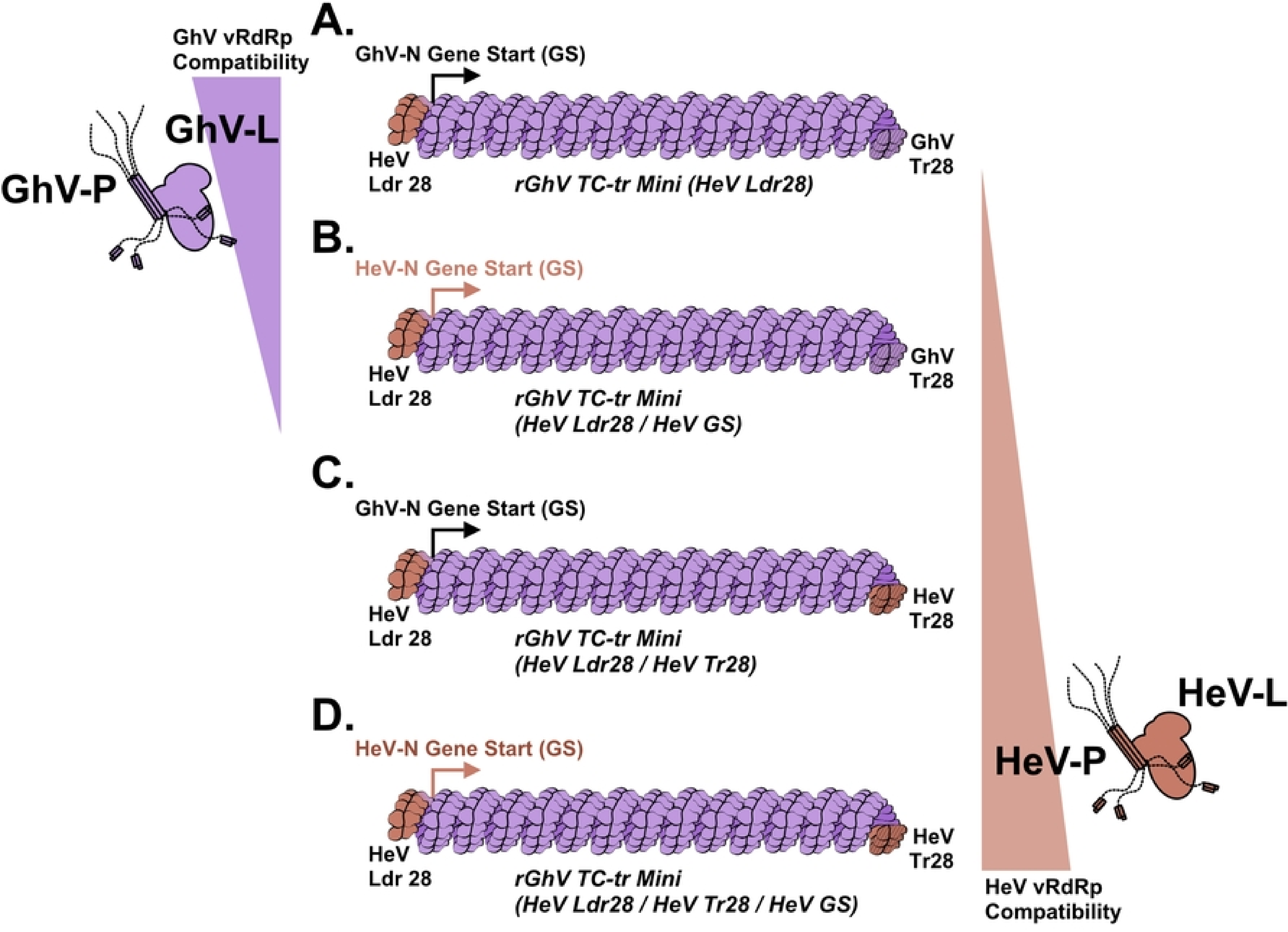

Supplementary Figure 5

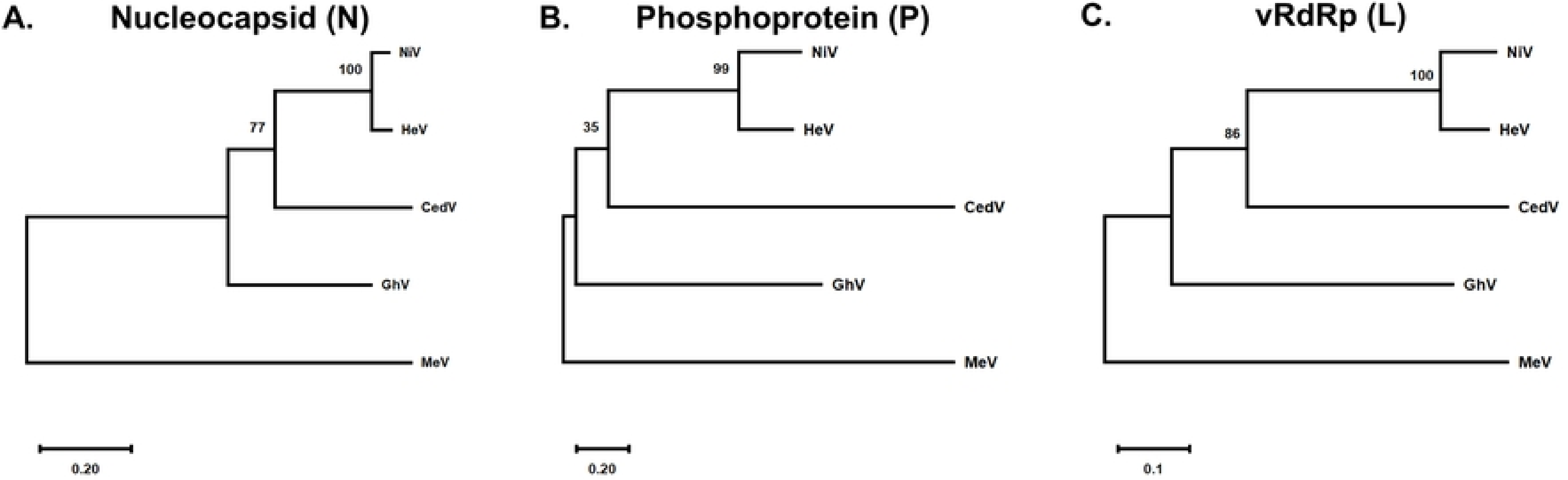

Supplementary Figure 6

```
    Hexamer     1      2      3      4      5

HeV Ldr28:   UGGCUU GUUCCC CUUUAU ACCUAU GCACAA
HeV  Tr28:   UGGCUU GUUCCC AUUUCU CUCUAG CAAUAA
             ****** ******  *** *   ***    * **

             1    6      12     18     24     30
```

Supplementary Figure 7
